# Cardiovascular Complications Are Increased in Inflammatory Bowel Disease: A Path Toward Achievement of a Personalized Risk Estimation

**DOI:** 10.3390/jpm15090418

**Published:** 2025-09-02

**Authors:** Vito Annese, Maria Laura Annunziata, Guglielmo Albertini Petroni, Emanuele Orlando, Sofia Cinque, Marzio Parisi, Paolo Biamonte, Giuseppe Dell’Anna, Anna Latiano, Serenella Castelvecchio

**Affiliations:** 1 Unit of Gastroenterology, I.R.C.C.S. San Donato Policlinic, San Donato Milanese, 20097 Milan, Italy; marialaura.annunziata@grupposandonato.it (M.L.A.); guglielmo.albertinipetroni@grupposandonato.it (G.A.P.); emanuele.orlando@grupposandonato.it (E.O.); sof.cinque@studenti.unina.it (S.C.); ignaziomarzio.parisi01@universitadipavia.it (M.P.); biamonte.paolo@hsr.it (P.B.); dellanna.giuseppe@hsr.it (G.D.); 2 Department of Gastroenterology, University Vita & Salute, San Raffaele, Via Olgettina 60, 20132 Milan, Italy; 3 Gastroenterology Unit, Department of Clinical Medicine and Surgery, University Federico II, Via Pansini 5, 80131 Naples, Italy; 4 Department of Internal Medicine and Medical Therapy, University of Pavia, Via Adolfo Ferrata 9, 27100 Pavia, Italy; 5 Division of Gastroenterology and Endoscopy, Fondazione IRCCS “Casa Sollievo della Sofferenza”, San Giovanni Rotondo, 71013 Foggia, Italy; a.latiano@operapadrepio.it; 6 Unit of Cardiovascular Prevention, I.R.C.C.S. San Donato Policlinic, San Donato Milanese, 20097 Milan, Italy; serenella.castelvecchio@grupposandonato.it

**Keywords:** inflammatory bowel disease, ulcerative colitis, Crohn’s disease, cardiovascular disease, cardiovascular risk, major cardiovascular events, multi-omics, artificial intelligence

## Abstract

**Background/Objectives**: The global burden of inflammatory bowel diseases (IBDs) continues to rise, with up to 50% of patients experiencing extraintestinal manifestations. Cardiovascular diseases (CVDs) are of particular concern, ranking as the second leading cause of mortality in this population. Despite a comparatively lower prevalence of traditional cardiovascular (CV) risk factors, the persistent inflammatory milieu and immune dysregulation inherent to IBD may contribute to heightened CVD risk. In this study, following a review of the current literature, an ongoing prospective trial designed to clarify CV risk profiles in IBD patients is detailed. **Methods**: A cohort of patients with IBD is being enrolled for comprehensive baseline evaluation of CV risk factors, lifestyle metrics, and disease characteristics. The incidence of major adverse cardiovascular events (MACEs) will be tracked and contrasted with a gender- and age-matched non-IBD cohort over a 2-year follow-up period. In cases of MACE occurrence, a multi-omics analysis—including genomic, proteomic, transcriptomic, and microbiome profiling—will be performed, along with a parallel evaluation in matched IBD controls without MACE. An artificial intelligence (AI) framework will support the analysis of this complex dataset. **Results**: To date, over 150 patients with IBD have been enrolled, and detailed phenotypic data and biological samples have been collected. **Conclusions**: We aim to introduce an IBD-specific correction factor for existing CV risk scores upon study completion. This is particularly relevant for individuals under 40 years of age, who are often inadequately assessed by current risk stratification models

## 1. Introduction

Crohn’s disease (CD) and ulcerative colitis (UC), the principal forms of inflammatory bowel disease (IBD), are showing a rising global prevalence, affecting an estimated 7 million individuals and placing a substantial strain on healthcare systems [1,2]. Many patients experience progressive intestinal damage, with complications such as hospitalization, surgery, malignancy, and elevated mortality risk [3,4]. Up to 50% of patients also develop extraintestinal manifestations, and there is an increased predisposition toward other immune-mediated disorders [5,6]. Among these comorbidities, cardiovascular disease (CVD) stands out in significance, especially as the aging IBD population sees a growing incidence of CVD-related mortality—now the second leading cause of death [7,8,9].

Despite advances in therapy, approximately one-third of patients with IBD exhibit primary non-response, and nearly half experience secondary loss of response [10]. While CVD remains the foremost cause of death globally [11], patients with IBD are more susceptible to premature atherosclerosis and myocardial infarction, particularly during active disease phases [12]. Alarmingly, conventional CV risk models often fail to capture this elevated risk, suggesting an underestimation in this specific population [13]. A comprehensive systematic review of more than 2 million patients with IBD identified smoking (24.2%) and alcohol use (4.6%) as the leading lifestyle risk factors. The common comorbidities included hypertension (30%), diabetes (14.4%), dyslipidemia (18.4%), pre-existing CVD (22%), and renal impairment (10%) [14].

### 1.1. IBD and CVD: A Shared Pathogenesis

IBD arises from a multifaceted interaction of genetic predisposition, immune dysregulation, environmental exposures, and dysbiosis of the gut microbiota—culminating in heightened cytokine activity [3,4]. This sustained inflammatory and immune-activated state may foster cardiovascular complications even in the absence of prominent conventional CV risk factors [7,8,12].

Emerging evidence points to shared pathophysiological mechanisms between IBD and CVD, including genetic overlap (e.g., *NOD2*, *CDKN2B*, *stromelysin*, and *ApoE* polymorphisms), shared environmental triggers like smoking, and convergent immune pathways [15]. Disruption of both intestinal epithelial and vascular endothelial barriers through cytokines like TNF-α may provide a mechanistic link between gut inflammation and vascular pathology [16]. In addition, gut microbiota alterations commonly seen in IBD have been implicated in the pathogenesis of cardiovascular conditions such as atherosclerosis [17].

Although the SCORE2 model developed by the European Society of Cardiology remains the prevailing tool for CV risk estimation, it has not been extensively validated in IBD populations and does not account for the heightened inflammatory burden, unlike the 1.5 correction factor applied in rheumatoid arthritis [18].

### 1.2. Risk of Major Adverse Cardiovascular Events in Inflammatory Bowel Disease

Multiple studies have demonstrated a significantly higher incidence of MACEs in IBD populations, with increases ranging from 25% to 300% compared with matched controls. A pivotal study by Bernstein et al. involving over 88,000 participants (8072 with IBD) reported an elevated incidence rate ratio (IRR) for ischemic heart disease (IHD) in patients with IBD (IRR: 1.26; 95% CI: 1.11–1.44) [19]. Similarly, a Danish cohort study analyzing 4.5 million individuals, including 28,833 with IBD, found a sharply elevated IHD risk in the first year after diagnosis (IRR: 2.13; 95% CI: 1.91–2.38), with a continued increased risk over time (IRR: 1.22; 95% CI: 1.14–1.30) [20]. A U.S. database study of over 29 million individuals showed a 25% higher likelihood of myocardial infarction among patients with IBD (OR: 1.25; 95% CI: 1.24–1.27) [21]. Standard CV risk algorithms may underestimate this burden, as chronic systemic inflammation is central to pathogenesis of CVD in IBD [22,23]. Elevated risks of stroke and cerebrovascular events have also been documented [24,25], with C-reactive protein (CRP) serving as a potential inflammatory marker [25]. A meta-analysis encompassing cohort and case–control studies confirmed a 21% increase in stroke risk in patients with IBD (OR/RR: 1.21; 95% CI: 1.08–1.34) [26]. Young IBD patients are particularly vulnerable to ischemic strokes, especially during active disease phases [24,25,26]. Additionally, an increased incidence of atrial fibrillation may further amplify the cerebrovascular risk [27].

### 1.3. Impact of IBD Treatments on Cardiovascular Risk

Certain IBD treatments, particularly corticosteroids, may worsen cardiovascular profiles by inducing hypertension, insulin resistance, and dyslipidemia [28]. However, steroid use often reflects severe disease activity, a known risk factor for CVD itself. Conversely, Rungoe et al. reported that patients with IBD treated with 5-ASA exhibited a reduced risk of IHD (IRR: 1.16 vs. 1.36 for non-users; *p* = 0.02), with prolonged use linked to even lower risks (IRR: 1.08) [20].

TNF-α inhibitors may provide cardiovascular protection [29]. A large observational study found a lower incidence of acute arterial events (AAEs) among TNF inhibitor users (HR: 0.79), with a similar risk reduction for recurrent events (HR: 0.75) [30]. Thiopurines have shown similar protective effects.

Data on the cardiovascular safety of newer biological therapies available, such as vedolizumab, ustekinumab, mirikizumab, and risankizumab, remain limited; however, no concerning safety signals related to AAEs have been identified thus far.

Janus kinase (JAK) inhibitors, including tofacitinib, filgotinib, and upadacitinib, are among the most recent agents approved for moderate to severe ulcerative colitis and, in the case of upadacitinib, also Crohn’s disease. Clinical trials have noted reversible elevations in serum lipids, including total cholesterol, LDL, and HDL, during early treatment with tofacitinib [31]. Hypercholesterolemia is a known contributor to cardiovascular events, and concerns have been raised regarding MACEs associated with this drug class. The ORAL Surveillance trial, which enrolled rheumatoid arthritis (RA) patients aged ≥50 with pre-existing cardiovascular risk factors, found a higher incidence of MACEs in those treated with tofacitinib (5 or 10 mg twice daily) compared with those on TNF inhibitors (HR: 1.33; 95% CI: 0.91–1.94) [32].

Based on recommendations from the Pharmacovigilance Risk Assessment Committee (PRAC), the European Medicines Agency’s Committee for Medicinal Products for Human Use (CHMP), has advised limiting the use of JAK inhibitors in patients with elevated cardiovascular risk, reserving them for cases with no appropriate alternatives. Nonetheless, a meta-analysis reviewing the safety of JAK inhibitors across IBD and other immune-mediated inflammatory diseases (IMIDs) reported 30 studies assessing MACEs in 32,765 treated individuals (17 on tofacitinib, 6 on upadacitinib, 4 on baricitinib, and 3 on filgotinib), with a pooled incidence rate of 0.67 per 100 patient-years. The relative risk of MACEs was estimated at 1.07 (95% CI: 0.56–2.03) in the analysis of 22 controlled trials [33].

The following tables illustrate

– Established cardiovascular risk factors (Table 1);
– Shared biological mechanisms that may drive IBD and cardiovascular disease (Table 1);
– Treatment-related cardiovascular risks (Table 2);
– Summary of key studies conducted on this topic (Appendix A) [34,35,36,37,38,39,40,41,42,43,44,45,46,47,48,49,50,51,52,53,54,55,56,57,58,59,60,61,62,63,64,65,66,67,68,69,70,71].

**Table 1:** Risk factors for IBD and cardiovascular diseases.

Traditional CV Risk Factors	Risk Factors for IBD	Shared Risk Factors for IBD and CVDs	Specific Risk Factors in IBD for CVDs
Obesity	Genetic factors	Dysbiosis	Inflammatory cytokines
Hypertension	Dysbiosis	Genetic factors	Oxidative stress
Age	Smoke (CD)	Smoke (CD)	Hypercoagulability
Smoke	Diet	? Diet	Endothelial dysfunction
Diabetes	? No breast feeding	? Stress	
Dyslipidemia	? Antibiotics abuse		
Family history of IHD or stroke	Family history of IBD		
? Stress			
? Diet			

IHD = ischemic heart disease; CD = Crohn’s disease. ? = conflicting data

**Table 2:** Impact of IBD therapies on main cardiovascular risk.

IBD Medications	Potential CV Effect
5-Aminosalicylic acid	↓ Inflammation, ↓ platelet activation ↓ CV complications
Corticosteroids	↑ CV Risk (? proxy of more severe IBD)
Thiopurines	↓ Inflammation, ↓ atherosclerosis and arterial events
Anti-TNFα	↓ Inflammation, ↓ atherosclerosis and arterial events
Vedolizumab	↓ Inflammation, limited data, no increased CV risk
Ustekinumab	↓ Inflammation, limited data, no increased CV risk
IL-23 inhibitors	↓ Inflammation, limited data, no increased CV risk
Anti-JAK	↓ Inflammation, ↑ CV risk in RA
SP1 Modulators	↓ Inflammation, limited data, no increased CV risk

RA = rheumatoid arthritis. ? = conflicting data. ↑ = increased. ↓ = reduced.

### 1.4. IBD and MACEs: Risk Prediction

There remains a critical need for robust assessment of MACE incidence in IBD, therapy-related risks and benefits, lifestyle influences, and disease activity (Appendix A) [34,35,36,37,38,39,40,41,42,43,44,45,46,47,48,49,50,51,52,53,54,55,56,57,58,59,60,61,62,63,64,65,66,67,68,69,70,71]. We have launched a prospective study involving a well-characterized IBD cohort, matched with controls by age and gender. Multi-omics profiling and AI-based analyses will be applied to refine traditional CV risk stratification and uncover novel biomarkers.

### 1.5. Dissecting the Complexity of CV Risk in IBD Patients

As summarized in the previous sections, there is still an unmet need to evaluate the real amount of CV risk in patients with IBD because of the lack of prospective trials, and the limitation of the available CV risk scores designed for an older background population compared to the patients with IBD. In addition, there is a lack of a systematic evaluation of other possible shared risk factors, such as genetic polymorphisms or dysbiosis, and more importantly, the need to evaluate the full systematic dataset with a hypothesis-free powerful methodology, such as AI-driven algorithms. For these reasons, an ongoing study designed to overcome these limitations is described here.

## 2. Materials and Methods

### 2.1. Study Population

Our first aim was to prospectively enroll a large cohort of IBD patients in the Unit of Gastroenterology of the Research Hospital San Donato Policlinic, San Donato Milanese (Mi), for 1 year to be compared with an age/gender-matched cohort under follow-up at the Unit of Cardiology of the same Institution. To this end, a set of specialized, privacy-preserving large language models (LLMs), coordinated by an agentic framework provided by Agora Labs [72], will be applied to different types of data sources containing records of patients under follow-up at the San Donato Policlinic to maximize enrollment. Specifically, the LLMs will extract clinical findings, diagnoses, disease activity indicators, family and social history items, past and current treatments, and other clinical data from all kinds of free-text notes. Under the clinicians’ supervision, such extracted data will drastically expand the underlying data pool and cohort size available for analysis. Data from both unstructured and structured sources will be harmonized to allow for multi-omics queries and model development, starting from descriptive statistics to the development of predictive algorithms. Following the signature of informed consent, all patients with confirmed IBD according with standard of care [3,4] will have a baseline evaluation of family and personal CVD risk factors with a standard questionnaire, including occurrence in family members of MACEs defined as ischemic heart disease (IHD), myocardial infarction (MI), stroke (ST), atrial fibrillation (AF), heart failure (HF), and thromboembolic events (TE). All patients will be evaluated with SCORE2 and the glyco-metabolic inflammatory axes. In case of the occurrence of a high CV risk score (>10 <20%), the carotid intima-media thickness (IMT) will be measured by Doppler ultrasound as a surrogate marker for subclinical atherosclerosis. In case of very high CV risk (more than 10% or more than 7.5% in subjects older than 69 years or younger than 50 years, respectively), the patient will be referred to the cardiologist (if not previously referred) for further investigations. To all patients, lifestyle modification measures will be given in written (i.e., stop smoking, minimize alcohol use, and follow a Mediterranean diet). Patients with IBD will undergo a careful baseline evaluation of disease activity and ongoing therapy, standard laboratory investigations, ileocolonoscopy, and biopsies as standard of care [3,4]. In addition, in all IBD patients, different biological materials (whole blood, plasma/serum, tissues, and stool samples) will be collected and stored in the Policlinic Biobank. All patients will then be followed for at least 2 years, and every year, a re-evaluation of IBD activity, ongoing therapy, lab tests, and monitoring of the occurrence of MACEs will be recorded. The enrollment of the study population will be performed according to the protocol approved by the local ethics committee (Milan 29/1/2025 CET 510-2024), which was recently also registered as NCT07095634.

The control cohort will be that of the CV-PREVITAL trial (a multicenter, prospective, randomized, controlled, open-label interventional trial—NCT05339841) designed to compare the effectiveness of an educational and motivational mobile health (mHealth) intervention versus usual care in reducing CV risk. The Cardiology Unit of IRCC Policlinico San Donato has already enrolled 1109 healthy individuals in this trial. These participants, who were free from known CVDs at baseline, have already completed a 1-year follow-up and are still under cardiological evaluation to monitor their CV health over time. The control group for the IBD cohort, matched for age and sex, will be extrapolated from this population.

### 2.2. Multi-Omics Evaluation

The second aim is to undertake an extensive multi-omics, metabolic, and histological evaluation.

Genomics: Germline genetic markers using DNA extracted from peripheral blood samples collected from patients with IBD will be evaluated. A panel of approximately 30 single-nucleotide polymorphisms (SNPs) will be analyzed using TaqMan allelic discrimination assays [73]. The list of SNPs to be evaluated will be selected by reviewing the available literature of genes involved in immune and inflammatory processes. Univariate and multivariate stepwise logistic regression models will be used to identify the variables associated with CVD risk and MACEs in patients with IBD.

Transcriptomics: Genome-wide expression profiling of mRNA will be conducted using RNAs extracted from tissue samples collected before and after treatment of selected patients to identify pivotal and critical genes associated with therapy response. The designed whole-transcript array used will provide the most accurate, sensitive, and comprehensive measurement of protein coding and long intergenic non-coding RNA transcripts [74]. Gene expression microarray analysis and functional enrichment analysis of the sample data will be performed to reveal abnormally expressed genes at the tissue level and to elucidate the biological significance of the differential expression pattern.

Metagenomics: A comprehensive sequencing of the gut microbiome will be performed. DNA will be extracted from stool samples at baseline and after 2 years of follow-up, and the variable V3 and V4 regions of the 16S rRNA gene will be sequenced. All procedures will follow extensively validated protocols to perform accurate taxonomic and functional profiling of the microbiome composition using newly developed custom bioinformatics and statistical tools [75].

Proteomics: To perform proteomic profiling of plasma samples at baseline and at the end of follow-up, cutting-edge technology will be applied to overcome the limitation of the outstanding dynamic range of plasma proteins [76]. With these targeted proteomic approaches, we will analyze the potential modulation of proteins involved in the most relevant physio-pathological pathways, including inflammation, immune response, and metabolic dysregulation.

Metabolic evaluation: In addition to the standard laboratory evaluation of the glyco-metabolic axis, patients with IBD will be assessed with Fibroscan at the time of enrollment and at the end of follow-up for the presence of hepatic steatosis.

Histology: The biopsy samples will be oriented on acetate cellulose filters, fixed in formalin, and embedded in paraffin. Sections stained with hematoxylin and eosin (H&E) will be used to evaluate the histological activity with standard validated scores. In addition, Claudin-2 immunohistochemistry will be performed on 4 um sections using the Claudin-2 antibody (Cell Signaling Technology, E1H90 clone, 1:100 dilution). The primary antibody will be detected using a biotin-free polymeric horseradish peroxidase (HRP)–linker antibody conjugate system with heat-induced epitope retrieval using the Bond III automated immune-stainer. Claudin-2 expression will be evaluated with a semi-quantified score along the cytoplasmatic cell membranes of the glandular epithelium and/or of the surface epithelium [77].

### 2.3. Prospective Evaluation

At the end of the 2-year follow-up, all patients in the IBD cohorts and the control population will be re-evaluated in terms of CVD risk using SCORE 2, the standard laboratory and cardiologic investigations will be repeated, and the occurrence of new MACEs will be reported. All patients with IBD will be regularly evaluated during the follow-up at 3–6 month intervals, and all will receive a clinical, endoscopic, and histologic evaluation at the end of follow-up. The subgroup of patients with the occurrence of MACEs and those with clinical and endoscopic remission will also repeat the transcriptomics and metagenomics evaluation.

### 2.4. Objectives of the Study

The primary objective of the study will be to evaluate the basal CVD risk in our IBD population compared with the age- and gender-matched cohort of non-IBD patients.

The secondary objectives will be as follows:

– Correlation of the basal CVD risk with multi-omics biomarkers, disease activity, lifestyle (i.e., diet, smoking) and therapy;
– To evaluate the occurrence of MACEs at the end of follow-up in the patients with IBD and control populations;
– To investigate the possible correlation between high CVD risk and MACE occurrence with multi-omics findings;
– To identify possible biomarkers of increased CVD risk compared with the control population;
– To establish possible correction factors to apply to the standard traditional CVD risk score to incorporate the IBD inflammatory burden.

### 2.5. Statistical Analysis

In this study, a combination of descriptive and inferential statistical methods will be required to ensure robust analysis. For descriptive statistics of the key characteristics of the study (CD, UC, and matched controls), we used the mean and standard deviation (for continuous variables such as age and BMI), median and interquartile range (for skewed variables), and counts and percentages (for categorical variables, such as sex and smoking status). To check the balance between the groups, the standardized mean differences (SMDs) will assess the balance of covariates between the groups. An SMD < 0.1 typically indicates good balance. *t*-tests or Mann–Whitney U tests (depending on normality) for continuous variables will compare means or medians across groups. Chi-square tests or Fisher’s exact tests for categorical variables will compare proportions across groups. In view of the evaluation of the outcome of MACEs, we will use Kaplan–Meier survival analysis to estimate the time to the cardiovascular event, stratified by subgroups. In addition, a Cox proportional hazard regression will allow for evaluation of the hazard ratio (HR) of MACEs while adjusting for confounders. The log-rank test will compare the survival curves between the different groups. We will also apply a multivariate logistic regression evaluating the outcome as binary (e.g., MACEs occurring vs. not occurring during the study period). To ensure the robustness of our findings, sensitivity analyses, such as propensity score matching sensitivity may be performed. Because missing data is common in prospective studies, multiple imputation methods will be used to handle missing data in covariates or outcomes.

The power of our sample was estimated by Cohen’s h effect size for proportions based on different hypotheses about the annual incidence rate of main MACEs in Italy (ISTAT—National Institute of Statistics), a range from 1% to 5% per year, and the estimated increase in cardiovascular events in the Crohn’s disease and ulcerative colitis groups, a range from 1.25 (25% increase) to 3.5 (250% increase). Under the assumption of an incidence rate of 1% per year in the control population, a sample size of 300 patients with IBD (CD and UC) and 300 matched controls will have 80% power to detect an HR of 2.5 or greater. Under the assumption of an incidence rate of 5% per year, there will be approximately 80% power to detect a hazard ratio equal to 1.6. The recruitment period was set to 1 year with 4 years of follow-up (2 years retrospective and 2 years prospective) for a total duration of 5 years. The significance level was set at 5%. A dropout of 5% in both groups is assumed. Effect size parameters: Follow-up period of 3 years retrospectively and 2 years prospectively; alpha (α) of 0.05 (standard significance level); power (1—β) of 90%.

For genomics, logistic regression and generalized linear models will be used for association analyses between the identified genetic variants and clinical outcomes, adjusting for confounders, such as age, sex, and disease duration.

Transcriptome data will be analyzed for differential expression patterns, with statistical significance assessed through empirical Bayes methods, followed by pathway enrichment analysis using gene set enrichment analysis (GSEA) and functional network mapping through tools such as STRING (consortium 2024) or Cytoscape (ver. 3.10.3).

Microbiome data will undergo community profiling through alpha and beta diversity metrics, and associations with clinical endpoints will be tested via multivariate analysis methods, such as canonical correspondence analysis (CCA) and redundancy analysis (RDA). Furthermore, metagenomic functional profiles will be reconstructed using PICRUSt2 or HUMAnN3, allowing for functional comparison across patient subgroups. Integration across omics layers will be realized through data integration analysis for biomarker discovery using latent components (DIABLO) and complemented by machine learning-driven feature selection techniques (e.g., LASSO, elastic-net).

Targeted proteomics will be analyzed by ANOVA and Tukey’s for a multiple comparison test. To evaluate the concordance of histological evaluation (active/remission) of different scoring system and Claudin 2 immunostaining, Cohen’s kappa (k) will be evaluated.

In addition, to carry out the systematic evaluation of the large, multidimensional dataset, overcoming the limitation of sample size and length of follow-up, and to detect unexpected pathogenetic hypotheses and biomarkers, an artificial intelligence model will be implemented [78]. This will be performed with the use of the Agora Labs platform. Agora^®^ Node supports the training of machine-learning and AI algorithms in a localized or federated architecture, thus allowing multivariate analysis to correlate clinical and multi-omics findings and other attributes included in the study, as well as deeper evaluation of multidimensional datasets by applying random forest (RF) and support vector machine (SVM) models, among others. Random forest (RF) and support vector machine (SVM) models, specifically, will be tested on feature selection tasks and on the identification of predictors of cardiovascular events from clinical and multi-omics data. RF will highlight influential factors (e.g., genetic markers, microbiome composition, and inflammatory markers), which will then be prioritized in subsequent modeling stages. SVMs, known for their performance with smaller datasets, will then refine classification based on selected features, creating a baseline model for cardiovascular risk. Neural networks, particularly deep neural networks (DNNs) or convolutional neural networks (CNNs), will also be tested in the handling of the study’s complex, high-dimensional dataset, especially in identifying non-linear relationships across genomics, transcriptomics, and proteomics data. After RF and SVM have highlighted the most predictive features, these will be integrated into neural networks to improve accuracy in predicting cardiovascular events.

## 3. Results

No solid results of the ongoing study are given; only the status of recruitment at the time of writing is reported. In total, 167 patients with IBD have been enrolled with detailed phenotyping and bio-sampling.

### 3.1. Characteristics of the IBD Study Population

The main characteristics of the IBD study population are depicted in Supplementary Table S1. Enrollment is still in progress, and the end of follow-up is estimated to be by the end of 2027. During the last 3–6 months of the project, multi-omics evaluation and analysis will be performed. At that time, the full data analysis will be shared.

### 3.2. Characteristics of the Control Population

Table 3 reports the basal characteristics of the control population enrolled in the study CV PREVITAL. The enrollment of this cohort is complete, and all subjects underwent evaluation of their CV risk score and provided blood samples at baseline to evaluate total cholesterol and its fractions, fasting glucose, glycate hemoglobin, triglycerides, homocysteine, and C reactive protein. In addition, bio-samples were collected for further possible multi-omics evaluation and carotid intima-media thickness (IMT) evaluation by Doppler ultrasound. By applying the propensity score from this cohort, an age- and gender-matched control population will be selected for comparison with the IBD cohort.

## 4. Discussion

The precise mechanisms responsible for the development of cardiovascular events in individuals with inflammatory bowel disease (IBD) remain incompletely defined [7,8,12]. Additional studies are needed to unravel the intricate relationship between systemic inflammation, immune system disturbances, and the onset of cardiovascular disease (CVD) in this population [8]. IBD represents a heterogeneous group of conditions with diverse clinical presentations and trajectories [3,4]. However, most existing research tends to treat IBD as a unified entity, often overlooking possible distinctions in CVD risk between ulcerative colitis (UC) and Crohn’s disease (CD), or across varying levels of disease severity. Further investigation is essential to better characterize these differences and to enable more tailored preventive and therapeutic strategies. Additionally, much of the current evidence is derived from observational or retrospective studies, limiting the ability to draw definitive causal inferences regarding CVD development in this setting (see Appendix A).

At present, there are no specific, validated guidelines for cardiovascular risk assessment in patients with IBD. Many IBD patients are younger adults who fall outside the demographic typically assessed by conventional cardiovascular risk calculators. Nevertheless, the 2019 guidelines from the European Society of Cardiology recognized IBD—alongside conditions such as cancer therapies, rheumatoid arthritis, and systemic lupus erythematosus—as requiring heightened vigilance in screening, counseling, and management for atherosclerotic disease [79]. Similarly, guidance from the European Alliance of Associations for Rheumatology highlights the importance of monitoring inflammatory activity using biomarkers, a principle that may be equally relevant to IBD populations [18].

A multidisciplinary approach is essential for appropriately addressing the interplay between IBD and cardiovascular risk. Beyond early detection and effective treatment, secondary prevention should be prioritized. In this context, recommendations from the American Heart Association support strategies to mitigate cardiovascular risk in patients with IBD, emphasizing the maintenance of disease remission as a key factor in reducing atherosclerotic cardiovascular disease (ASCVD) risk [11]. These guidelines also underscore the need for aggressive control of modifiable risk factors, including routine screening of blood pressure, blood glucose, lipid profiles, and promotion of healthier lifestyle choices. Moreover, there is a strong rationale for adopting personalized prevention strategies in IBD care. Clinicians can tailor interventions by identifying individuals at greater risk of cardiovascular complications, thereby facilitating informed, shared decision-making [11].

Several limitations still exist in fully understanding the IBD–CVD relationship, particularly with regard to therapeutic effects, biological pathways, and the availability of laboratory tools, such as biomarkers, that could help anticipate cardiovascular risk and pathogenesis.

Traditional risk calculators, such as SCORE2, likely underestimate CVD risk in patients with IBD. As seen in rheumatoid arthritis, a correction factor of 1.5 is recommended to account for the persistent inflammatory burden; a similar adjustment may be warranted in patients with IBD [18].

Our study will provide a comprehensive analysis of how disease activity, treatment regimens, and multi-omics profiles influence cardiovascular risk. This approach may help identify common risk pathways and open new prevention and management avenues. At the moment, we have only reported the status of recruitment in Section 3. The control population inclusion, derived from the PREVITAL study, is complete and will be used for gender- and age-matched comparison by applying the propensity score methodology. In contrast, the enrollment of the patients with IBD will be completed by the end of 2025, and after 2 years of follow-up, multi-omics evaluation will be performed and analyzed.

In Italy, there are currently an estimated 250,000 to 300,000 patients with IBD, with projections indicating a possible doubling over the next decade [1]. This trend will inevitably place greater pressure on the national healthcare system. A major strength of our study is its structured approach—applying standard care protocols and monitoring both clinical disease features and lifestyle data over a 5-year period (3 years retrospectively, 2 years prospectively). In addition, we will compare this cohort to a well-matched, rigorously followed control population. Multi-omics analysis will be used to identify potential biomarkers associated with increased cardiovascular risk.

Upon completion of the study, we aim to propose an IBD-specific correction factor to enhance the accuracy of existing CV risk scoring systems. This will be particularly valuable for younger patients—especially those under 40 years—who are not adequately captured by current tools.

Indeed, this subgroup appears to show a more pronounced disparity in MACE incidence compared to the general population. Notably, this will be the first prospective investigation in Italy specifically designed to evaluate CVD risk in patients with IBD and one of only a few worldwide [43]. The findings of this study will offer important insights to clinicians, researchers, and policymakers, contributing to more informed strategies to reduce cardiovascular morbidity and mortality in this vulnerable group.

## 5. Conclusions

The exact underlying mechanisms of cardiovascular events in IBD remain poorly understood. More research is required to elucidate the complex interplay between inflammation, immune dysregulation, and CVD pathogenesis in IBD, which encompasses a spectrum of diseases with varying clinical phenotypes and courses. More studies are needed to understand these nuances and to guide targeted interventions. Existing studies primarily rely on observational or retrospective data, making it difficult to establish definite causal relationships between specific factors and CVD development. In this study, all consecutive patients with IBD will be evaluated for their basal SCORE 2 and complete disease characteristics and will undergo screening cardiologic investigations to assess potential cardiovascular issues. Following at least 2 years of follow-up, the whole cardiologic and gastroenterological assessment will be repeated, and the occurrence of MACEs will be investigated and correlated with the multi-omics assessment and control cohort. This in-depth evaluation will potentially reveal unexpected and silent underlying high risks for MACEs and direct the subjects, under the control of a cardiologist, to make significant changes in their lifestyle, diet, and therapy. More importantly, the multi-omics evaluation may reveal biomarkers of increased CV risk and suggest corrective factors for the standard CV risk score in this patient population.

## Figures and Tables

**Table 3:** Basal characteristics of the control population (mean ± SD).

	Overall (N = 1109)	Male (N = 492)	Female (N = 617)
Age (yrs)	55.9 ± 6.5	56.9 ± 6.5	55.1 ± 6.2
BMI	25.7 ± 4.5	26.8 ± 3.9	24.9 ± 4.8
Waist (cm)	90.3 ± 13.4	98.1 ± 11.4	84.5 ± 12.9

## Data Availability

The original contributions presented in this study are included in the article. Further inquiries can be directed to the corresponding author.

## Supplementary Materials

The following supporting information can be downloaded at: https://www.mdpi.com/article/10.3390/jpm15090418/s1, Table S1: Characteristics of the enrolled IBD study population so far (mean ± SD).

## Abbreviations

The following abbreviations are used in this manuscript:

IBD	Inflammatory bowel disease
CD	Crohn’s disease
UC	Ulcerative colitis
CV	Cardiovascular
CVD	Cardiovascular disease
MACE	Major cardiovascular event
AI	Artificial intelligence
IHD	Ischemic heart disease
MI	Myocardial infarction
AF	Atrial fibrillation
AAE	Acute arterial events

## Appendix A. List of More Relevant Studies Investigating the Risk of MACEs in IBD

**Authors (Ref.)**	**Year**	**Study Design**	**Population**	**Results**
Bernstein et al. [19]	2008	Population-based	8060 IBD vs. 80,489 controls	RR IHD 1.26 (95% CI 1.11–1.44)
Rungoe et al. [20]	2008	Population-based	28,833 IBD vs. 4,570,820 non-IBD	RR IHD 2.13 (95% CI 1.91–2.38) in the first year after diagnosis
Andershon et al. [25]	2010	Population-based	8054 CD and 161,078 controls	Increased OR for IS in <50 years 2.93 (95% CI 1.44–5.98)
Osterman et al. [34]	2011	Retrospective	15,498 UC and 9829 CD vs. 237,592 controls	No increased risk of MI
Yarur et al. [35]	2011	Longitudinal	356 IBD vs. 712 controls	OR CAD 4.08 (95% CI 2.49–6.70)
Haapamaki et al. [36]	2011	Retrospective	2831 IBD vs. 5662 controls	OR for CHD 1.88 (95% CI 1.29–2.73); active disease risk factor (*p* = 0.018)
Sridhar et al. [37]	2011	Cross-sectional	Inpatient database	OR for dysrhythmias in females aged 18–39 years 2.05 (95% CI 1.71–2.44)
Kristensen et al. [38]	2013	Population-based	20,795 IBD vs. 199,978 controls	RR for MI 1.17 (95% CI 1.05–1.31); rate increased during flares (1.49; 95% CI 1.16–1.93)
Aggarwal et al. [39]	2014	Retrospective	131 IBD with CAD vs. 524 controls	IBD younger, less active smokers, and lower BMI (*p* = 0.03)
Tsai et al. [40]	2014	Population-based	11,822 IBD vs. 47,288 controls	HR for ACS 1.72 (95% CI 1.53–1.94)
Huang et al. [41]	2014	Retrospective	18,392 IBD vs. 73,568 controls	RR for IS 1.12 (95% CI 1.02–1.230)
Kristensen et al. [27]	2014	Population-based	24,499 IBD vs. 236,275 controls	IRR for stroke during active disease (1.57; 95% CI 1.32–2.21) and chronic activity (1.71; 95% CI 1.32–2.21)
Singh et al. [42]	2014	Meta-analysis	98,240 IBD	OR for CVA 1.18
Kristensen et al. [43]	2014	Prospective	23,681 IBD vs. 5,412,966 controls	IRR for hospitalization for HF 1.37
Xiao et al. [26]	2015	Meta-analysis	124,493 IBD and 4748 stroke	RR of stroke in IBD 1.29 (95% CI 1.16–1.43); increased risk in younger patients
Yuan et al. [44]	2015	Meta-analysis	8 cohorts	RR of stroke in IBD 1.32 (95% CI 1.20–1.44)
Keller et al. [41]	2015	Population-based	3309 CD vs. 13,236 controls	HR for stroke 1.91 (95% CI 1.65–2.22)
Barnes et al. [45]	2016	Retrospective	567,438 IBD with MI vs. 78,121,000 population	IBD had a lower rate of hospitalization OR 0.51 (95% CI 0.50–0.52)
Feng et al. [46]	2017	Meta-analysis	177,330 IBD vs. 5,573,620 controls	RR IHD 1.24 (95% CI 1.14–1.35)
Sun et al. [47]	2018	Meta-analysis	27 studies	RR stroke 1.25 (95% CI: 1.08, 1.44), RR IHD 1.12 (95% CI: 1.05, 1.21), RR MI 1.17 (95% CI: 1.05, 1.21)
Aniwan et al. [35]	2018	Longitudinal	736 IBD vs. 1472 controls	HR MI 2.82 (95% CI 1.98–4.04)
Kirchgesner et al. [48]	2018	Population-based	210,162 IBD	SIR for AAEs 1.19 (95% CI 1.16–1.22); SIR for IHD 1.17 (95% CI 1.13–1.21); highest risk < 55 years and disease active
Le Gall et al. [49]	2018	Case–control	30 IBD with AAEs vs. 60 matched IBD without	Disease activity associated with AAEs OR 10.4 (95% CI 2.1–49.9)
Panwhar et al. [21]	2019	Population-based	290,430 IBD vs. 28,799,790 control	OR for MI 1.25 (95% CI 1.24–1.27)
Aarestrup et al. [50]	2019	Population-based	1293 IBD vs. 107,496 controls	More frequent CVD 13.2% vs. 10.9% (*p* = 0.009)
Choi et al. [51]	2019	Population-based	37,477 IBD vs. 112,431 controls	HR for MI 1.8 (95% CI 1.47–2.21); age < 40 years HR 2.96 (95% CI 1.96–4.47)
Kumar et al. [52]	2019	Longitudinal	2449 IBD, 271 IBD with HF vs. 20,444 HF	HR for HF with complication in IBD 1.67; IRR of HF 2.54 (95% CI 2.13–3.04) during flare
Ghoneim et al. [53]	2020	Population-based	261,890 IBD vs. 51,914,660 control	RR for CVA OR 8.07 (95% CI 7.9–8.2)
Zui et al. [54]	2020	Meta-analysis	62,287 IBD vs. 367,337 controls	OR for AF 2.26
Biondi et al. [23]	2020	Case–control	52 IBD vs. 37 controls	Carotid intima–media thickness greater (0.690.12 mm vs. 0.630.12 mm, *p* = 0.031); atherosclerotic carotid plaque 25% vs. 5.4% *p* = 0.032
Li et al. [55]	2021	Meta-analysis	710,520 IBD vs. 5,671,535 controls	RR IHD 1.26 (95% CI 1.2–1.32)
Gill et al. [56]	2021	Observational	15,292 IBD vs. 30,584 controls	No increased risk of MI (HR 1.05 (95% CI 0.89–1.23)
Card et al. [57]	2021	Retrospective	31,175 IBD vs. 154,412 controls	HR for MI 1.83 (95% CI 1.28–2.62) for acute disease and chronic activity (HR 1.69; 95% CI 1.24–2.3)
Chen et al. [24]	2021	Meta-analysis	149,908 patients with stroke	RR for stroke in IBD 1.21 (95% CI 1.08–1.34)
Tanislav et al. [58]	2021	Retrospective	11,497 IBD vs. 11,497 controls	HR stroke 1.5; HR for TIA 1.93
Lee et al. [59]	2021	Cross-sectional	157,085 IBD	Premature ASCVD OR 1.07; extremely premature OR 1.61
Pemmasani et al. [60]	2021	Cross-sectional	24,220 IBD vs. 6,872,415 controls	Mortality for ACS in IBD OR 0.81
Setyawan et al. [61]	2022	Retrospective	34,687 IBD vs. 34,687 non-IBD	No increased risk of MI [IRR 0.62 (95% CI 0.44–0.88]
Nasir et al. [62]	2022	Retrospective	951 IBD vs. 165 ASCVD	RR for ASCVD 1.58 (95% CI 1.17–2.13)
Fang et al. [63]	2022	Retrospective	1425 IBD vs. 1588 controls	Higher risk for IHD (12.1% vs. 5.5%; *p* < 0.001); higher risk < 35 years (CD OR = 6.3; UC OR = 3.35)
Alayo et al. [22]	2023	Retrospective	5094 IBD vs. 20,376 controls	HR for AAEs 1.19 (95% CI 1.08–1.31)
D’Ascenzo F et al. [64]	2023	Meta-analysis	515,455 controls and 77,140 IBD	HR for MI 1.36 [1.12–1.64], death 1.55 [1.27–1.90] other CV disease, such as stroke (HR 1.22 [1.01–1.49]
Sleutjes JAM et al. [65]	2023	Case–control	235 IBD, 829 controls	OR 2.01, (95% CI 1.23–3.27) for CVDs
Pam HN et al. [66]	2024	Nationwide	CDC database	Age-adjusted mortality rates (AAMR) decreased from 0.11 in 1999 to 0.07 in 2020
Sun J et al. [67]	2024	Nationwide	81,749 IBD, 382,190 controls	Higher risk of HF HR 1.19, 95% CI 1.15–1.23
Gardezi SA et al. [68]	2025	Administrative data	41,635 deaths due to CVDs in IBD	Increased age-adjusted mortality rates (AAMRs) from 1999 to 2023 (*p* = 0.0004)
Ebrahimi R et al. [69]	2025	Matched cohort	987 IBD and 9571 controls	Increased MACEs HR 1.37 (95% CI, 1.24–1.52).
Luo C et al. [70]	2025	Meta-analysis	2,802,955	Higher risk of stroke, HR of 1.30 [95% CI 1.21–1.39].
Cohen-Heyman et al. [71]	2025	Retrospective	14,768 IBD and 120,338 controls	IHD associated with IBD in males (HR = 1.82; 95% CI: 1.52–2.17)
RR = relative risk; IRR = incidence rate ratio; OR = odds ratio; SIR = standardized incidence ratio; HR = hazard ratio; IHD = ischemic heart disease; IS = ischemic stroke; MI = myocardial infarction; CAD = coronary artery disease; CHD = coronary ischemic disease; ACS = acute coronary syndrome; CVA = cardiovascular atherosclerosis; AAE = acute artery events; HF = heart failure; AF = atrial fibrillation; MACEs = major cardiovascular events; AAMR = age-adjusted mortality rate; ASCVD = atherosclerotic cardiovascular disease; TIA = transient ischemic attack; CVA = cardiovascular accident (stroke).				
