# Supplementary material for: Cardiovascular Complications Are Increased in Inflammatory Bowel Disease: A Path Toward Achievement of a Personalized Risk Estimation
Source: J Pers Med. 2025 Sep 2;15(9):418. doi: 10.3390/jpm15090418 (PMC12471200; doi:10.3390/jpm15090418)
Supplement: Supplementary file 1 [file jpm-15-00418-s001.zip › jpm-3800709-supplementary.pdf]

Table S1. Characteristics of the enrolled IBD study population so far (mean±SD)

	**CD (n=91)**	**UC (n=76)**
Age (years)	34.7±14.3	36±13.8
Duration from diagnosis (years)	17±11	16±10
Therapy at the time of enrollment	-	-
5-ASA	15	52
Steroids	31	18
IMS (Azt/Mtx)	16	6
Biologics	77	45
Small molecules	-	4
Activity of the disease at the enrollment (yes/no)	57/34	43/33
Previous surgery (yes/no)	39	3
BMI > 25 (yes/no)	5	4
Smoking (yes/no/ex)	31/36/2	26/38/3
Family history of MACEs (yes/no)	2	3
Personal history of MACEs (yes/no)	3	2
Diabetes (yes/no)	1	1
EIMs (yes/no)	36	22
High cholesterol (yes/no)	15	12
Samples for the Biobank (yes/no)	91	76
